# The Royal College of Psychiatrists should become British, not Royal

**DOI:** 10.1192/bjb.2024.97

**Published:** 2025-08

**Authors:** David Curtis

**Affiliations:** University College London, London, UK

**Keywords:** Monarchy, Royal College of Psychiatrists, MRCPsych, equality, diversity

## Abstract

The royal charter of the Royal College of Psychiatrists is generally taken to enhance its status. However, the concept of a hereditary monarchy is intellectually indefensible and the realities of the British monarchy exacerbate inequalities in the UK. The connection is particularly problematic for psychiatrists because of their role in the compulsory detention and treatment of patients. The Royal affiliation can only serve to emphasise the power inequalities in society associated with these activities. College members should feel free to discuss whether this situation should continue or whether we should be British rather than Royal.

In 1926 the Medico-Psychological Association received its royal charter to become the Royal Medico-Psychological Association, subsequently becoming the Royal College of Psychiatrists in 1971 through a supplemental charter.^1^ Many would view this affiliation to the British monarchy as conferring kudos, enhancing the reputation of psychiatry and putting it on a par with other medical specialties, which have their own Royal Colleges. Here, I argue that the association of the Royal College of Psychiatrists with a hereditary monarchy in fact goes against much of what psychiatry stands for and is harmful to the profession, to patients and to the country. It should become an aspiration of members for the College to rebrand as the British College of Psychiatrists.

## The concept of a hereditary monarchy is intellectually indefensible

Arguments in favour of the monarchy include emotional ones, such as those appealing to tradition or a general affection for the royal family, or pragmatic ones, such as those claiming (whether accurately or not) that the royal family contributes to Britain's tourist industry or that royal patronage increases the profile of charities. However, from a purely intellectual point of view it does not really seem possible to argue that it is a good thing that a single individual, based purely on accident of birth, should have conferred on them a status that provides them with a life of privilege, albeit alongside concomitant obligations. From all the understanding we have as psychiatrists of medicine, society and the human condition it simply makes no sense that somebody should be treated in this way.

Nor can we be comfortable with the idea that, however monarchy is attained, the monarch is widely acknowledged to be different from, and indeed superior to, all their compatriots. This is not necessarily about hard power but rather about status. The monarch is not an autocrat who can demand obedience, but it is certainly the case that deference to the monarch from their subjects is expected. When the monarch enters the room people will bow and curtsey and provide them with dutiful attention. The notion that the monarch is in some way a superior form of human being is not one that most psychiatrists would seek to defend.

## Monarchy contradicts notions of equality and diversity

There is no prospect that in our lifetimes we will see a monarch, who embodies the core of what it means to be British, who is not White, Anglican and, barring an unlikely set of circumstances, male. How can we promote the idea that all Britons are equal, and equally British, when the best of them will always be a White Anglican? The relationship of the Crown with the Church goes in two directions. On the one hand, the monarch is the head of the Church of England. On the other hand, the status of Charles as King was confirmed by a Christian religious ceremony, the coronation. In our pluralistic society we might accept that other people's religious practices have value and meaning for them and we might respect those practices accordingly. However, to acknowledge that Charles is our King is to affirm that this is the result of a Christian ceremony, involving anointing him with holy oil, consecrated in Jerusalem, in ‘a sacred moment between the Sovereign and God’.^2^ This would seem to put Anglicanism in a special category not only as the established religion of England but also with the capability to elevate the monarch above us, their subjects. The coronation ceremony is not akin to the swearing in process which might be undertaken by holders of office in Britain and abroad but rather a Christian religious rite which, we are supposed to believe, firmly and permanently establishes the monarch as our superior. Surely such a notion cannot coexist with ideas that human beings in Britain, along with their religions, cultures and ethnicities, are diverse but broadly speaking equal, each to be valued for themselves?

## It is damaging for society for medical colleges to support the monarchy

Although it is the case that some concepts might find widespread support throughout society, such as justice, welfare and democracy, unfortunately the monarchy cannot be listed among these. A considerable proportion of the population does still support the monarchy, but these numbers are falling with time and, for example, recent UK polling shows that more people under 50 would be in favour of an elected head of state rather than a monarch.^3^ However, the existence of the Royal Society, the Royal Academy and the medical Royal Colleges provides the monarchy with the stamp of approval of the country's finest scientists, artists and doctors. The Royal affiliation of these institutions sends the message that the monarchy is a good thing. Failing to challenge this situation means that we are taking a side on an issue which in reality divides the country. The College would, quite rightly, hesitate to take a position on one side or the other of any number of similarly contentious issues, but until now seems to have taken for granted the notion that solidarity with the monarchy is unproblematic. The fact that all the country's intellectual elites appear to be united on this issue sends a powerful message that the topic of whether or not the monarchy should continue to exist is not up for serious discussion in British society today.

## Affiliation with the monarchy undermines our relationship with patients

Psychiatrists are painfully aware of the inequalities within society, particularly those relating to mental health and perhaps most especially the extraordinarily high rates of psychotic illness and compulsory treatment among men of Black ethnicities.^4–6^ While the reasons for this are not fully understood and are likely to be multifactorial, both an effect of and contributor to higher rates of compulsory treatment may be a view of psychiatric services as being rooted in social structures that disadvantage Black people, with psychiatry being experienced as a further form of oppression, provoking resistance and fear.^6,7^ Although there is not a one-to-one equivalence between holding the MRCPsych qualification and being section 12 approved or being qualified to be a responsible medical officer, it is nevertheless true that this qualification will confer much of the legitimacy to detain and/or treat a patient against their will. Although the connection may not be explicit or conscious, it is not difficult to envisage that the R in the post-nominal represents a concrete reminder that not all Britons are equal and that power structures within society are very far from balanced. Is there not a possibility that the inherent, unavoidable anguish of the scenario might be at least slightly mitigated if psychiatrists were happy to accept the more modest MBCPsych instead?

## Conclusion

The College's endorsement of the system of hereditary monarchy should at least be a subject for debate. It would be entirely reasonable to take the view that the concept is irrational and outdated. If there is marked diversity of opinion among members, who cannot otherwise practise as psychiatrists, then it does not seem ethical to insist that the name of the College should implicitly support an institution which not all approve of. The College has an opportunity to display courage and leadership, for other medical specialties and for the country at large, by considering the option of renouncing its Royal status.

## Data Availability

Data availability is not applicable to this article as no new data were created or analysed in this study.

## About the author

David Curtis, MD, PhD, FRCPsych, is a retired consultant psychiatrist and an honorary professor in the UCL Genetics Institute, University College London, London, UK.

## References

[1] Bewley T. Madness to Mental Illness: A History of the Royal College of Psychiatrists. RCPsych Publications, 2008.

[2] Royal Household. The Anointing Screen. Royal Household, 2023 (https://www.royal.uk/news-and-activity/2023-04-29/the-anointingscreen).

[3] YouGov. YouGov / Republic Survey Result Fieldwork: 15th – 16th January. YouGov, 2024 (https://d3nkl3psvxxpe9.cloudfront.net/documents/Republic_Monarchy_240116_W.pdf).

[4] Halvorsrud K, Nazroo J, Otis M, Brown Hajdukova E, Bhui K. Ethnic inequalities in the incidence of diagnosis of severe mental illness in England: a systematic review and new meta-analyses for non-affective and affective psychoses. Soc Psychiatry Psychiatr Epidemiol 2019; 54: 1311–23. 31482194 10.1007/s00127-019-01758-y

[5] Barnett P, Mackay E, Matthews H, Gate R, Greenwood H, Ariyo K, et al. Ethnic variations in compulsory detention under the Mental Health Act: a systematic review and meta-analysis of international data. Lancet Psychiatry 2019; 6: 305–17. 30846354 10.1016/S2215-0366(19)30027-6 PMC6494977

[6] Lawrence V, McCombie C, Nikolakopoulos G, Morgan C. Navigating the mental health system: narratives of identity and recovery among people with psychosis across ethnic groups. Soc Sci Med 2021; 279: 113981. 10.1016/j.socscimed.2021.113981 33991793

[7] Lawrence V, McCombie C, Nikolakopoulos G, Morgan C. Ethnicity and power in the mental health system: experiences of white British and black Caribbean people with psychosis. Epidemiol Psychiatr Sci 2021; 30: e12. 10.1017/S2045796020001043 PMC8057456 33543688

